# A Critical Role of Fatty Acid Binding Protein 4 and 5 (FABP4/5) in the Systemic Response to Fasting

**DOI:** 10.1371/journal.pone.0079386

**Published:** 2013-11-14

**Authors:** Mas Rizky A. A. Syamsunarno, Tatsuya Iso, Hirofumi Hanaoka, Aiko Yamaguchi, Masaru Obokata, Norimichi Koitabashi, Kosaku Goto, Takako Hishiki, Yoshiko Nagahata, Hiroki Matsui, Motoaki Sano, Masaki Kobayashi, Osamu Kikuchi, Tsutomu Sasaki, Kazuhisa Maeda, Masami Murakami, Tadahiro Kitamura, Makoto Suematsu, YoshitoTsushima, Keigo Endo, Gökhan S. Hotamisligil, Masahiko Kurabayashi

**Affiliations:** 1 Department of Medicine and Biological Science, Gunma University Graduate School of Medicine, Maebashi, Gunma, Japan; 2 Education and Research Support Center, Gunma University Graduate School of Medicine, Maebashi, Gunma, Japan; 3 Department of Bioimaging Information Analysis, Gunma University Graduate School of Medicine, Maebashi, Gunma, Japan; 4 Department of Diagnostic Radiology and Nuclear Medicine, Gunma University Graduate School of Medicine, Maebashi, Gunma, Japan; 5 Department of Clinical Laboratory Medicine, Gunma University Graduate School of Medicine, Maebashi, Gunma, Japan; 6 Department of Laboratory Sciences, Gunma University Graduate School of Health Sciences, Maebashi, Gunma, Japan; 7 Metabolic Signal Research Center, Institute for Molecular and Cellular Regulation, Gunma University, Maebashi, Gunma, Japan; 8 Department of Biochemistry, Keio University School of Medicine, Shinjuku-ku, Tokyo, Japan; 9 JST, ERATO, Suematsu Gas Biology Project, Keio University School of Medicine, Shinjuku-ku, Tokyo, Japan; 10 Department of Cardiology, Keio University School of Medicine, Shinjuku-ku, Tokyo, Japan; 11 Department of Genetics and Complex Diseases and Nutrition, Broad Institute of Harvard and MIT, Harvard School of Public Health, Boston, Massachusetts, United States of America; University of Texas Health Science Center at Houston, United States of America

## Abstract

During prolonged fasting, fatty acid (FA) released from adipose tissue is a major energy source for peripheral tissues, including the heart, skeletal muscle and liver. We recently showed that FA binding protein 4 (FABP4) and FABP5, which are abundantly expressed in adipocytes and macrophages, are prominently expressed in capillary endothelial cells in the heart and skeletal muscle. In addition, mice deficient for both FABP4 and FABP5 (FABP4/5 DKO mice) exhibited defective uptake of FA with compensatory up-regulation of glucose consumption in these tissues during fasting. Here we showed that deletion of FABP4/5 resulted in a marked perturbation of metabolism in response to prolonged fasting, including hyperketotic hypoglycemia and hepatic steatosis. Blood glucose levels were reduced, whereas the levels of non-esterified FA (NEFA) and ketone bodies were markedly increased during fasting. In addition, the uptake of the ^125^I-BMIPP FA analogue in the DKO livers was markedly increased after fasting. Consistent with an increased influx of NEFA into the liver, DKO mice showed marked hepatic steatosis after a 48-hr fast. Although gluconeogenesis was observed shortly after fasting, the substrates for gluconeogenesis were reduced during prolonged fasting, resulting in insufficient gluconeogenesis and enhanced hypoglycemia. These metabolic responses to prolonged fasting in DKO mice were readily reversed by re-feeding. Taken together, these data strongly suggested that a maladaptive response to fasting in DKO mice occurred as a result of an increased influx of NEFA into the liver and pronounced hypoglycemia. Together with our previous study, the metabolic consequence found in the present study is likely to be attributed to an impairment of FA uptake in the heart and skeletal muscle. Thus, our data provided evidence that peripheral uptake of FA via capillary endothelial FABP4/5 is crucial for systemic metabolism and may establish FABP4/5 as potentially novel targets for the modulation of energy homeostasis.

## Introduction

Mammals have evolved a metabolic response system that enables them to survive for longer periods of food deprivation. The overall metabolic response to fasting operates at numerous levels and has been relatively well characterized [1]–[5]. In the fasted state, most tissues, except the brain and red blood cells, rely heavily on the direct utilization of fatty acids (FA) to generate energy. Prolonged fasting promotes the hydrolysis of triacylglycerol (TG) in adipose tissue, thereby increasing the concentration of non-esterified FA (NEFA) in plasma. Circulating FAs are taken up by the liver, where NEFA is either re-esterified to TG and secreted as very low density lipoprotein (VLDL), oxidized to synthesize adenosine triphosphate (ATP) in the mitochondria via β-oxidation or converted into ketone bodies that are used by many tissues, including the brain, during starvation.

Cytoplasmic fatty-acid-binding proteins (FABPs) actively facilitate the transport of lipids to specific compartments in cells. FABP4 (also known as aP2/ALBP/A-FABP) and FABP5 (also referred to as mal1/E-FABP) play important roles in the pathogenesis of metabolic diseases. Although mice deficient for either FABP4 or FABP5 demonstrate a modest phenotype due to their redundant function [6], [7], mice lacking both *Fabp4* and *Fabp5* (DKO mice) displayed a dramatic phenotype in their metabolism, including robust protection against diet-induced obesity, insulin resistance, type 2 diabetes, atherosclerosis, and fatty liver disease [8], [9]. This phenotype has been attributed to several potential mechanisms. First, the predominant feature of the metabolic phenotype is related to adipocyte FABPs with a more modest contribution from macrophages [10]. In addition, the increased supply of adipose tissue palmitoleate (C16∶1n7, also termed lipokine) or the increase in the ratio of shorter chain FAs (C14) to the longer chain (C18) in adipose and muscle tissues may also be candidates to mediate the improvement of insulin sensitivity and protection from fatty livers [9], [11]. Immunohistochemical analyses by several groups revealed that both FABP4/5 are also expressed in capillary endothelial cells in various organs, including the heart, skeletal muscle and adipose tissue [12], [13], suggesting a role of FABP4/5 in FA transport across capillary endothelial cells in these tissues. Very recently, we showed that DKO mice exhibited defective trans-endothelial FA transport with remarkable glucose uptake in the heart and red skeletal muscle in DKO mice during fasting, which was independent of insulin [14].

In this study, we showed that deletion of FABP4/5 resulted in a marked perturbation of metabolism in response to prolonged fasting, including hyperketotic hypoglycemia and prominent hepatic steatosis. Importantly, our data suggested that hepatic steatosis occurred not as a result of reduced FA oxidation (FAO), but rather from an increased influx of NEFA, which was likely attributed to an impairment in FA uptake in the heart and skeletal muscle in DKO mice.

## Results

### Blood Glucose is Decreased while NEFA and Ketone Bodies are Increased during Prolonged Fasting

**Figure 1:**
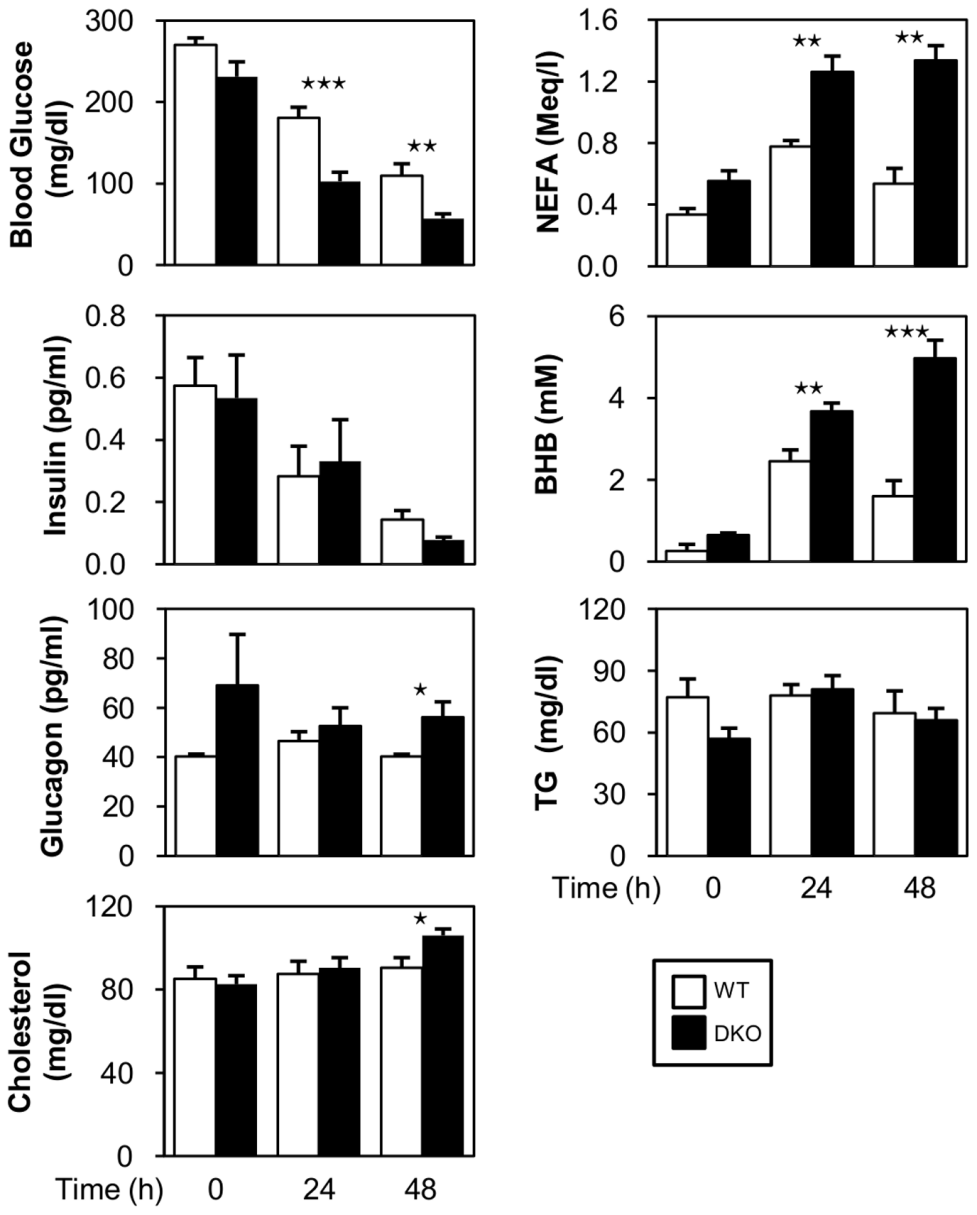
Blood glucose is decreased while serum levels of FFA and ketone bodies are markedly increased during prolonged fasting in DKO mice. Blood was collected from the vena cava inferior before (0-hr) and after fasting (24- and 48-hr) to measure blood glucose, NEFA, insulin, ketone bodies (BHB), triacylglycerol (TG) and cholesterol. n = 7−11/group. Blood was sampled from the retro-orbital plexus for plasma glucagon. n = 6/group. Data are shown as the mean ± SE. WT vs. DKO. ⋆p<0.05, ⋆⋆p<0.01, ⋆⋆⋆p<0.001.

We first studied several biochemical parameters of serum levels (figure 1). Glucose levels were significantly lower in DKO mice compared to wild-type (WT) mice after a 24- and 48-hr fast without a significant difference in the insulin level. The serum level of NEFA was also reciprocally increased in DKO mice, which was consistent with a lower uptake of FA by peripheral tissues during fasting [14]. In parallel with NEFA, the serum level of ketone body (β-hydroxybutyrate, BHB), a final product of ketogenesis, was enhanced in DKO mice as the fasting progressed. The serum glucagon level was also higher in DKO mice, which may function against lower glucose levels and promote lipolysis. TG and total cholesterol were comparable before and after fasting.

**Figure 2:**
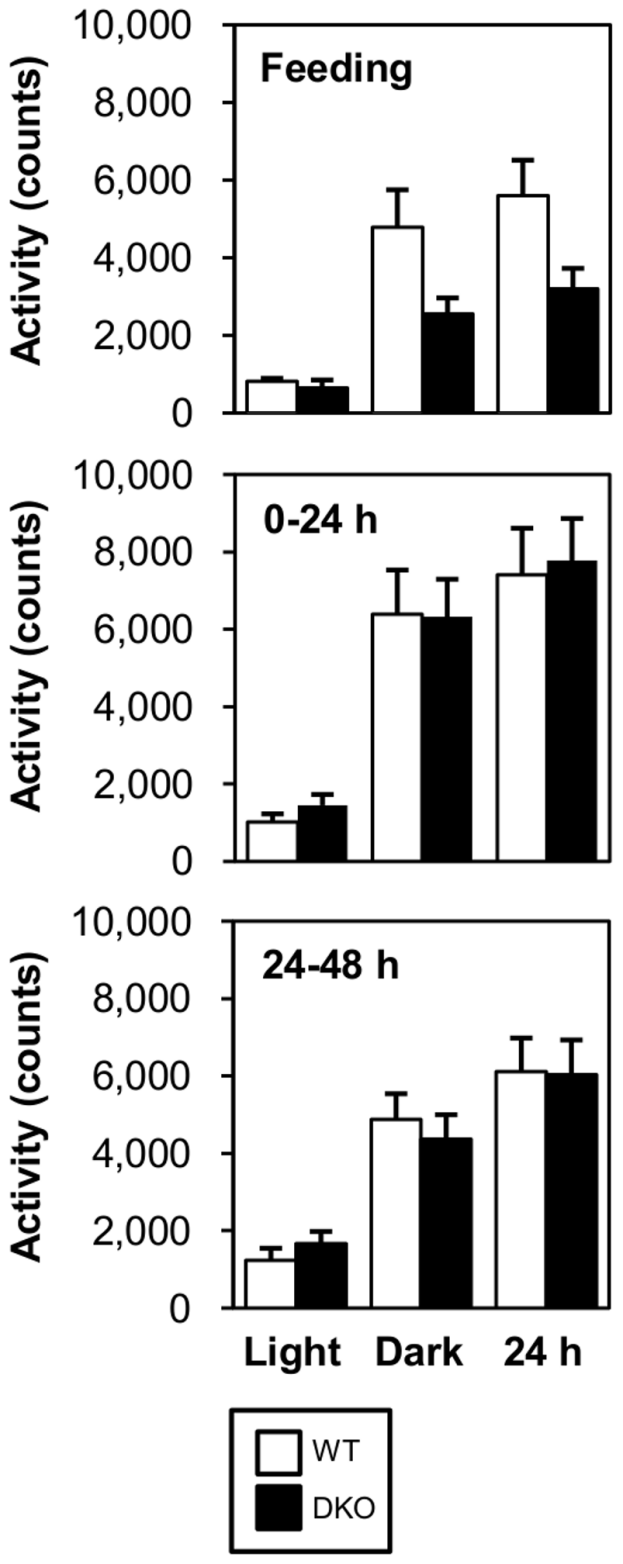
Locomotor activity is comparable between WT and DKO mice. Locomotor activity was measured during feeding and fasting (0–24 and 24–48 hr). Data are presented for 12 hours of the light phase, 12 hours the dark phase and combined light and dark phase (24 hr).

Despite extreme hypoglycemia in DKO mice, their physical activity was not reduced. Indeed, the locomotor activity was comparable between WT and DKO mice during prolonged fasting, although it tended to be reduced in the fed state (figure 2). We assumed that this conserved physical activity was attributed to a higher level of ketone bodies that could be utilized in many organs, including the brain, heart and skeletal muscle.

### Prolonged Fasting Causes Prominent Fatty Livers

**Figure 3.TG:**
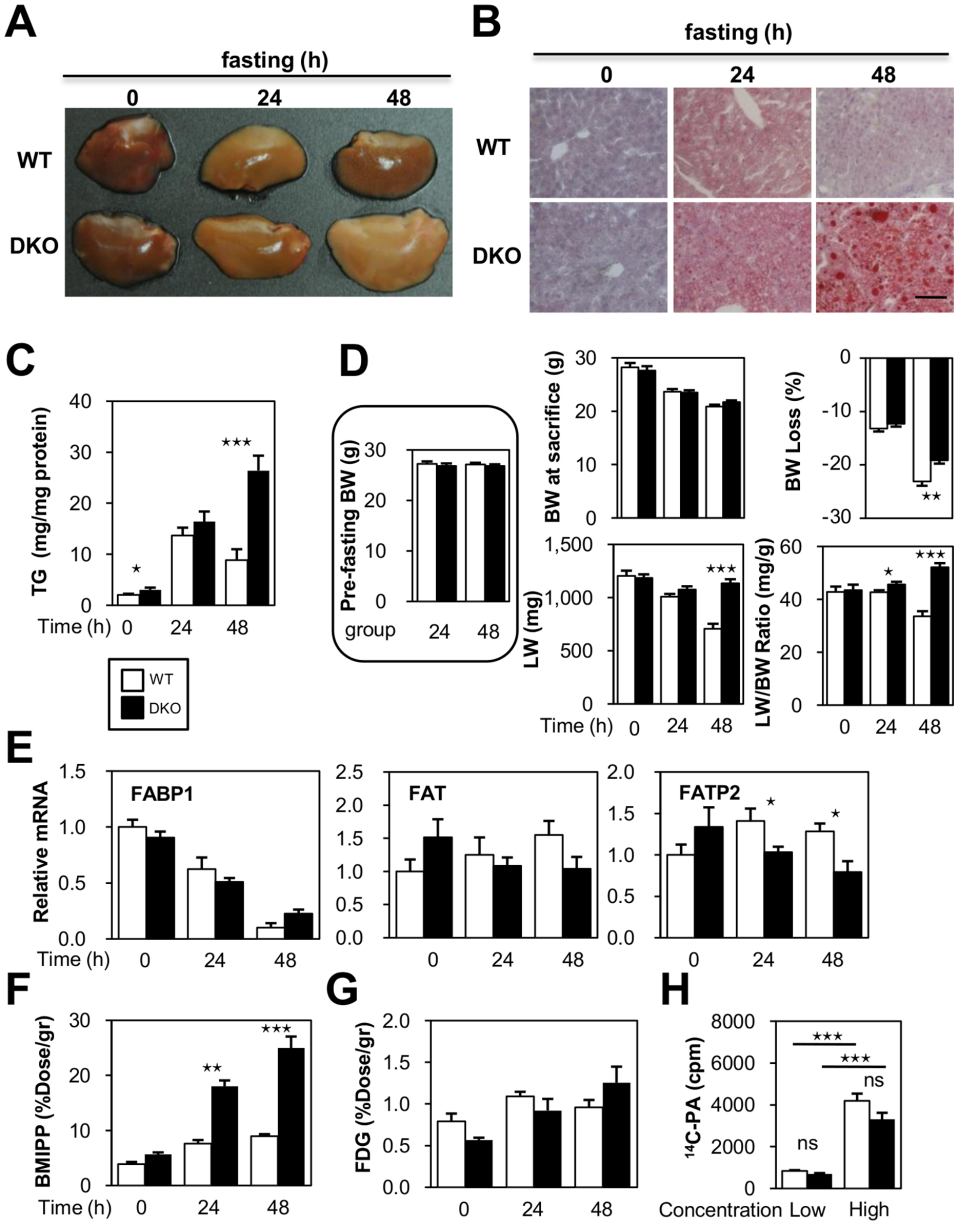
accumulation is enhanced in DKO livers during prolonged fasting. (A to E) Livers were isolated before (0 hour) and after fasting (24- and 48-hr). (A) Representative gross appearance of the liver. (B) Oil red O staining. Scale bar = 100 µm. (C) Triglyceride content in the liver (mg/mg protein). (D) Body weight (BW), % reduction in BW after fasting, liver weight (LW), ratio of LW relative to BW (LW/BW, mg/g). n = 11/group. (E) The expression of genes involved in FA uptake was determined using quantitative real-time PCR. (F and G) Mice received intravenous injections of ^125^I-BMIPP (5 kBq) and ^18^F-FDG (100 kBq) via the lateral tail vein before (0-hr) and after fasting (24- and 48-hr). The animals were sacrificed at 2 hours after injection. Uptake of ^125^I-BMIPP (F) and ^18^F-FDG (G) by the liver was quantified using a well-type gamma counter (n = 4). (H) Hepatocytes were isolated from WT and DKO livers. Uptake of ^14^C-palmitic acid (^14^C-PA) by hepatocytes was measured using a liquid scintillation counter. Note that uptake of ^14^C-PA was comparable between WT and FABP4/5 DKO hepatocytes. In addition, the uptake was proportional to the loading dose of ^14^C-PA. n = 6/group. Data are shown as the mean ± SE. WT vs. DKO. ⋆p<0.05, ⋆⋆p<0.01, ⋆⋆⋆p<0.001, ns =  not significant.

While hepatic TG accumulation did not appear to significantly differ between WT and DKO mice after a 24-hr fast, DKO mice exhibited prominent TG accumulation in the liver after a 48-hr fast (figure 3A, 3B and 3C). Although body weight was decreased in a time-dependent manner, liver weight was maintained in DKO mice, resulting in a higher ratio of liver weight to body weight (Figure 3D). In addition, there was a slightly smaller reduction in body weight in DKO mice after a 48-hr fast (figure 3D). Importantly, FABP4/5 expression was very low in the WT liver and was hardly detected in the sinusoidal endothelium using immunohistochemisty [14]. The expression of other genes associated with FA uptake in the liver, such as FABP1 and FAT (fatty acid translocase)/CD36, did not change in DKO mice compared to WT, although the expression level of FATP2 (fatty acid transport protein 2) was reduced after a 24- and 48-hr fast (figure 3E). These findings suggested that inducible expression of the genes relevant to FA uptake did not contribute to a large increase in FA uptake by the liver in DKO mice.

We next examined FA uptake in the liver during prolonged fasting using the FA analogue ^125^I-BMIPP. As expected, the uptake of ^125^I-BMIPP was markedly up-regulated after a 24- and 48-hr fast without a significant effecton^18^F-FDG uptake (figure 3F and 3G). Hepatocytes isolated from WT and DKO mice demonstrated a comparable ability to take up ^14^C-palmitic acid in a dose-dependent manner (figure 3H).

Taken together, these findings suggested that prolonged fasting triggered abundant lipid accumulation in the liver, which was not due to compensatory gene regulation by FABP4/5 deletion, but rather due to an increase in serum NEFA released from peripheral adipose tissue.

### Reduced VLDL Secretion Enhances TG Deposits in the Liver in DKOs

**Figure 4:**
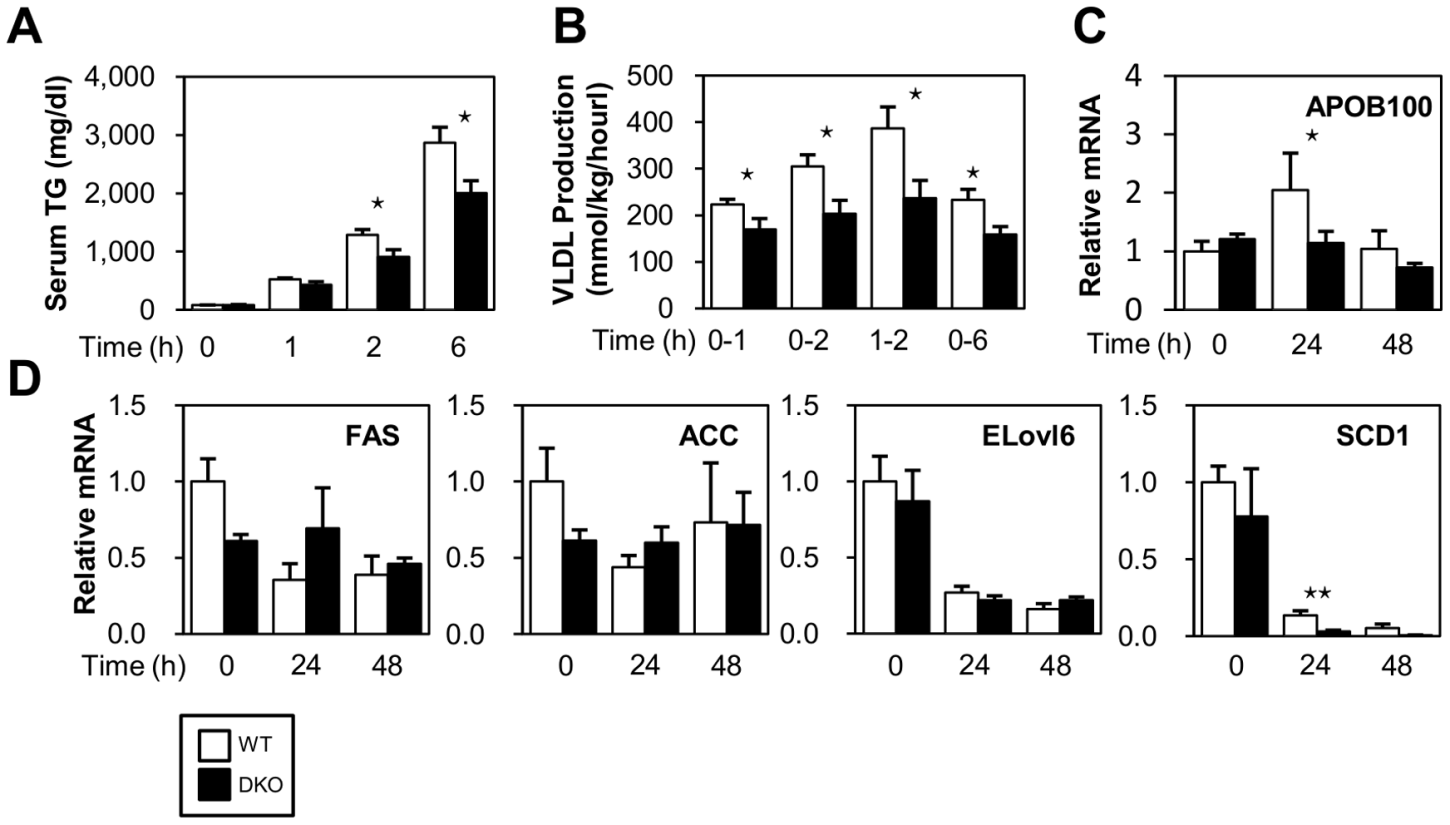
VLDL secretion is reduced in DKO mice after fasting. (A) Serum levels of TG were measured at the indicated time points after an intravenous injection of triton WR 1339 (500 mg/kg). (B) The VLDL production rate (mg/kg/hour) was calculated from the TG concentration of (A). n = 5−8/group. (C) The expression of ApoB100 was determined using quantitative real-time PCR. n = 4/group. Data are shown as the mean ± SE.WT vs. DKO. ⋆p<0.05. (D) The expression of genes involved in FA synthesis was determined using quantitative real-time PCR.

Very low density lipoprotein (VLDL) is assembled in hepatocytes and is directly secreted into the circulation to supply energy to extra hepatic tissues [15]. Thus, we determined the VLDL production rate by measuring temporal increases in plasma TG under conditions where TG hydrolysis by lipoprotein lipase (LPL) was inhibited. Interestingly, TG export was reduced in DKO mice during fasting (Figure 4A and 4B) despite the enhanced uptake of NEFA (Figure 3F). This result was concomitant with a lower expression of genes associated with VLDL formation, such as Apo-B100, in the liver of DKO mice (Figure 4C). Thus, the reduced secretion of VLDL from the liver could enhance pronounced fatty liver.

FA synthesis was generally suppressed during fasting. Consistent with this finding, the expression of mRNA for ELOVL6 and SCD1, which are enzymes involved in FA synthesis, were markedly reduced after a 24- and 48-hr fast in both WT and DKO mice (figure 4D). The expression of mRNA for FAS and ACC was not significantly different between WT and DKO mice. In DKO mice, the expression of SCD1 was significantly lower compared to WT mice after a 24-hr fast, suggesting that lipid accumulation in the DKO liver was not due to enhanced FA synthesis.

### Materials Associated with FAO are Decreased in the Livers of DKO Mice

**Figure 5:**
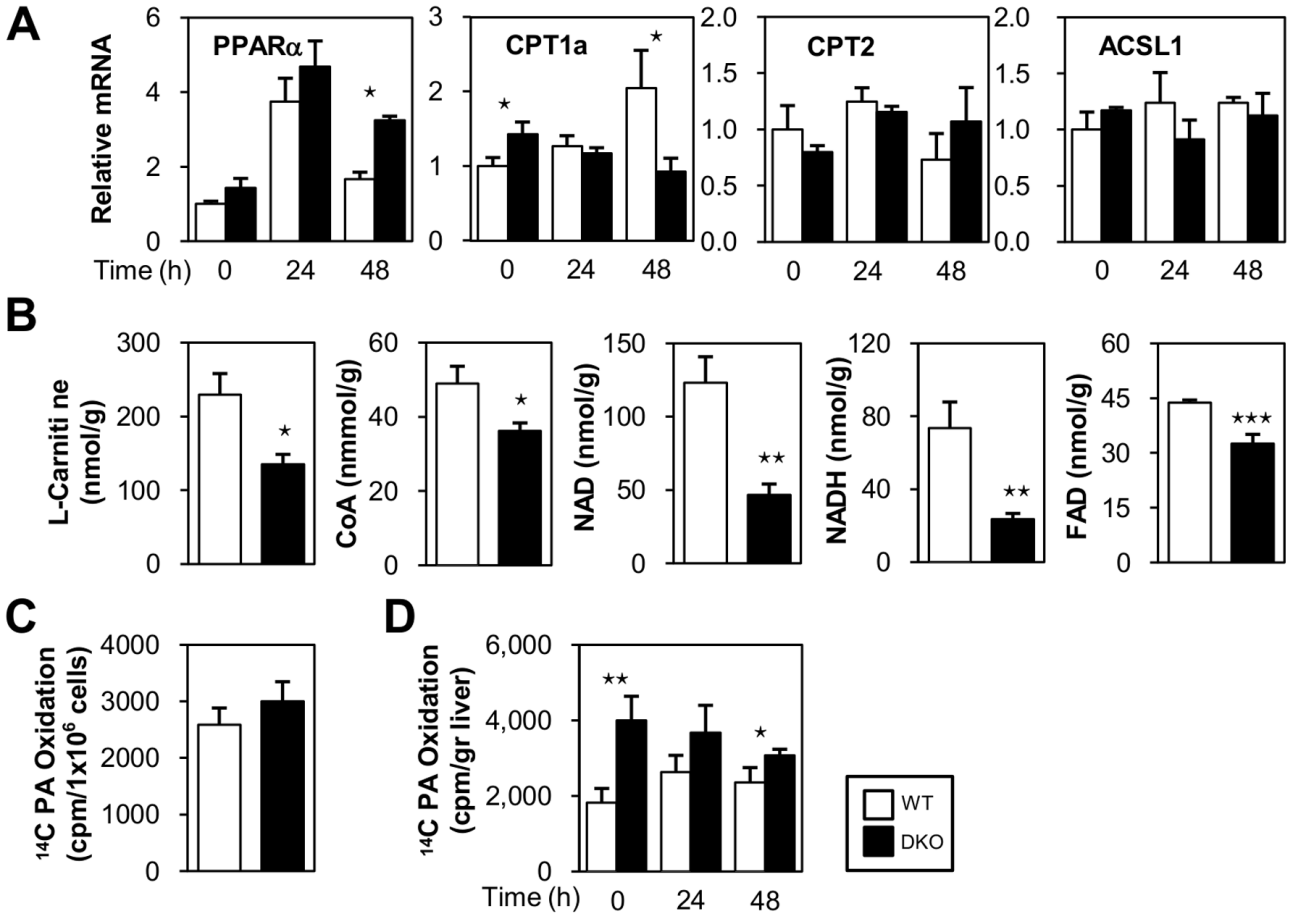
Rate of FA oxidation is enhanced in DKO mice. (A and B) Livers were isolated before (0 hour) and after fasting (24- and 48-hr). (A) The expression of genes involved in FA metabolism was determined using quantitative real-time PCR. (B) The indicated metabolites from the livers of WT and DKO mice after 48 hr fast were measured using metabolome analysis. n = 7/group. Note that energy charge ((ATP+1/2ADP)/(ATP+ADP+AMP) = 0.51, more than 0.5 guarantees sampling quality) served as a quality control of material handlings [32], [33]. The lactate/pyruvate ratio was higher compared to previous reports [34], which might be due to technological differences between the enzymatic/chemical approach used in previous studies and the CE-MS approach used in this study. (C) Estimation of FAO using primary cultures of hepatocytes. n = 10/group. (D) Estimation of FAO using liver homogenates before (0-hr) and after fasting (24- and 48-hr). n = 6/group. Data are shown as the mean ± SE. WT vs. DKO. ⋆p<0.05, ⋆⋆p<0.01, ⋆⋆⋆p<0.001.

Hepatic fat accumulation was also accelerated when FAO was disrupted [1]–[5]. Thus, we next examined the expression levels of the genes regulating FAO. The expression levels of carnitinepalmitoyltransferase 1a (CPT1a), a key enzyme for acyl-CoA transport into mitochondria, were lower in DKO livers despite the high expression of peroxisome proliferator-activated receptor α (PPARα) after a 48-hr fast (Figure 5A). The expression levels of CPT2 and acyl-CoA synthetase long-chain family member 1 (ACSL1), an enzyme that converts FA into acyl-CoA, were comparable between WT and DKO livers. Metabolome analysis revealed that L-carnitine, a carrier for acyl groups in the mitochondrial membrane, and Coenzyme A (CoA), an essential coenzyme for shuttling acyl groups and β-oxidation, were lower in DKO livers (figure 5B), suggesting that FA transport into the mitochondria and subsequent β-oxidation declined during fasting. Consistent with the decreased levels of L-carnitine and CoA, substrates for both L-carnitine (lysine and methionine) and CoA (cysteine) were significantly reduced (Table 1). NAD (nicotinamide adenosine dinucleotide), an indispensable coenzyme for β-oxidation, was also markedly reduced (figure 5B). Furthermore, its energy-rich reduced form NADH was also decreased (figure 5B). Consistent with this finding, tryptophan, a substrate for NAD, decreased during fasting (table 1). Flavin adenine dinucleotide (FAD), another coenzyme required for β-oxidation, was also reduced (figure 5B). Taken together, these findings suggested that NEFA taken up in the liver could be converted into acyl-CoA, although the reduced amount of other substrates, such as CPT1a, L-carnitine, CoA, NAD, and FAD, might limit the subsequent catabolism of FAs during prolonged fasting.

**Table 1:** Metabolome analyses of DKO livers.

Amino Acid	WT	FABP4/5 DKO	
**Glucogenic**			**Non essential**
Alanine	524.1±296.2	185.3±45.6*	
Arginine	3.7±0.6	2.1±0.5***	
Asparagine	80.6±19.1	28.9±5.8***	
Aspartate	305.0±44.3	247.9±65.2	
Cysteine	5.8±3.8	1.9±2.1*	
Glutamate	1130.9±231.8	1842.1±308.7***	
Glutamine	1455.1±410.0	1963.4±715.4	
Glycine	784.3±249.3	887.9±264.6	
Proline	84.7±16.7	60.2±11.9**	
Serine	344.2±136.3	189.4±62.8*	
**Glucogenic and Ketogenic**			
Tyrosine	43.9±9.7	30.4±10.2*	
**Glucogenic**			**Essential**
Histidine	230.8±37.6	288.5±68.3	
Methionine	14.0±5.9	6.7±0.5*	
Threonine	102.1±37.3	61.3±18.4*	
Valine	330.5±91.9	196.4±65.6**	
**Glucogenic and Ketogenic**			
Isoleucine	111.6±32.0	70.5±27.0*	
Phenylalanine	60.6±14.4	46.3±10.1	
Tryptophan	13.9±2.9	10.2±3.0*	
**Ketogenic**			
Leucine	246.4±73.0	196.8±70.3	
Lysine	254.8±81.3	135.5±35.3**	

(nmol/g weight).

Indicated metabolites from the livers of WT and DKO mice after 48-hour of fasting were measured using capillary electrophoresis-mass spectrometry (n = 7).

Data are shown as the mean ± SE. WT vs. DKO.

* p<0.05, **p<0.01, ***p<0.001.

### Capacity to Oxidize FA is Enhanced in DKO Livers

We next estimated the capacity of hepatocytes to oxidize FA using primary cultures and liver extracts. Both hepatocytes from WT and DKO mice showed a comparable FAO capacity, suggesting that the basal capacity of FAO in DKO hepatocytes was conserved (figure 5C). Unexpectedly, the estimated FAO rate in vitro using liver extracts prior to fasting was higher in the DKO compared to WT mice (figure 5D). A higher FAO in DKO extracts was also observed after a 48-hr fast despite a marked reduction in crucial cofactors, such as L-carnitine, CoA, NAD, and FAD, although it tended to decrease in a time-dependent manner after fasting (figure 5D). Because FA oxidation in the liver is regulated by multiple factors, such as fasting and the levels of glucose and glucagon [16], [17], the difference in ^14^C-palmitic acid oxidation between isolated hepatocytes and liver homogenates might be due to such circulating factors.

### Hepatic Steatosis by Prolonged Fasting is Reversible by Re-feeding in DKO Mice

**Figure 6:**
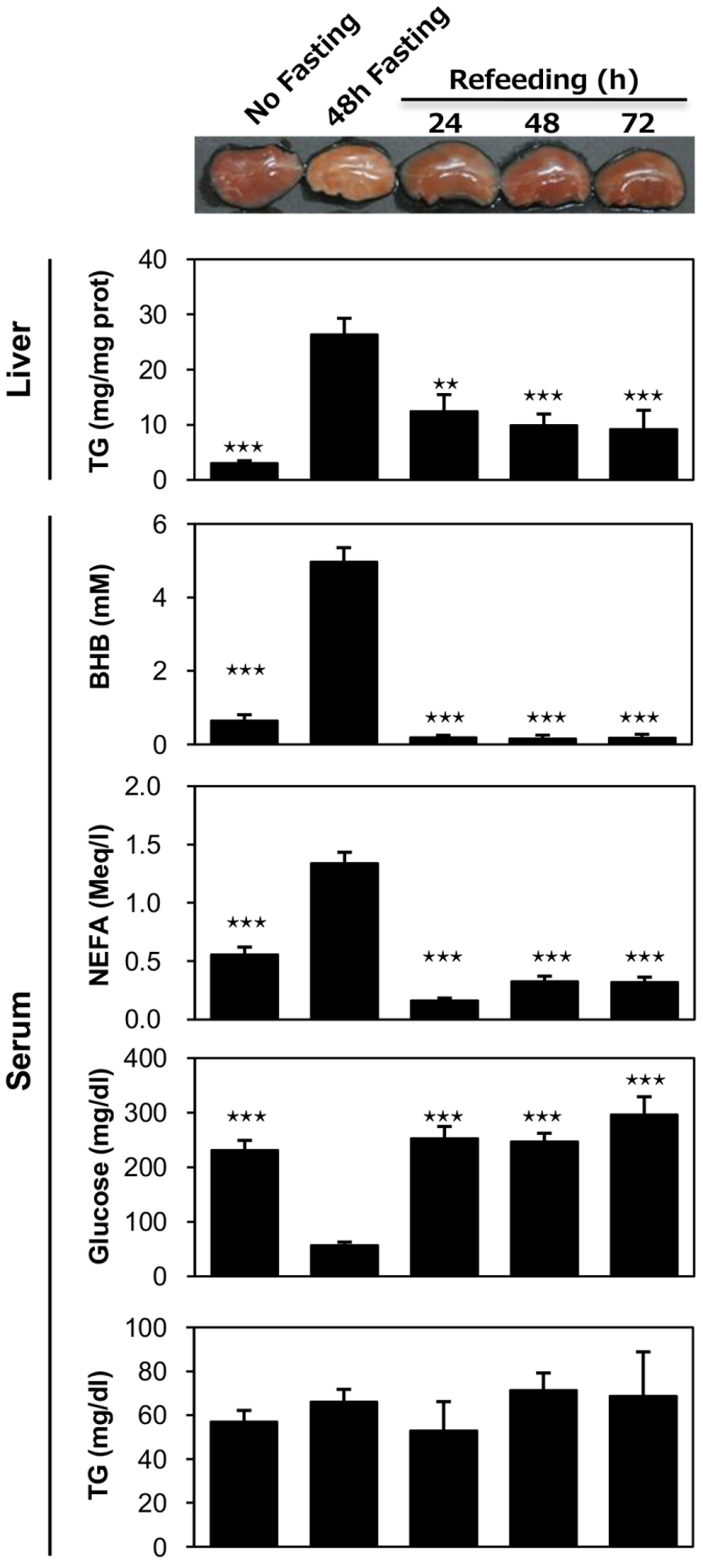
Fasting-induced hepatic steatosis is reversible. (A) DKO mice fasted for 48 hours. After resuming food intake, blood and liver samples were collected at the indicated time points (24-, 48- or 72-hr after refeeding). Mice that did not undergo fasting (0-hr) or experienced 48 hours of fasting were used as controls. The TG content in the liver and the serum levels of biochemical parameters (NEFA, ketone bodies, TG and glucose) were measured as previously described in the Materials and Methods section. n = 5−11/group. Data are shown as the mean ± SE. Control vs. no fasting/refeeding ⋆⋆p<0.01, ⋆⋆⋆p<0.001.

To determine the reversibility of fasting-induced hepatic steatosis in DKO mice, mice were re-fed after 48-hour of fasting (Figure 6). The liver color returned to a reddish color after one day of re-feeding. The TG content in the liver also decreased to nearly 50% on the first day after re-feeding. In addition, the serum levels of BHB and NEFA were dramatically decreased after re-feeding while the blood glucose returned to levels that were equivalent to normally fed mice. These findings were consistent with our assumption that prominent fatty livers in DKO mice after prolonged fasting was enhanced by an increased level of serum NEFA. Moreover, TG deposits in the liver were reduced when the NEFA level was markedly decreased by re-feeding.

### Impaired Glucose Homeostasis in DKO Mice

**Figure 7:**
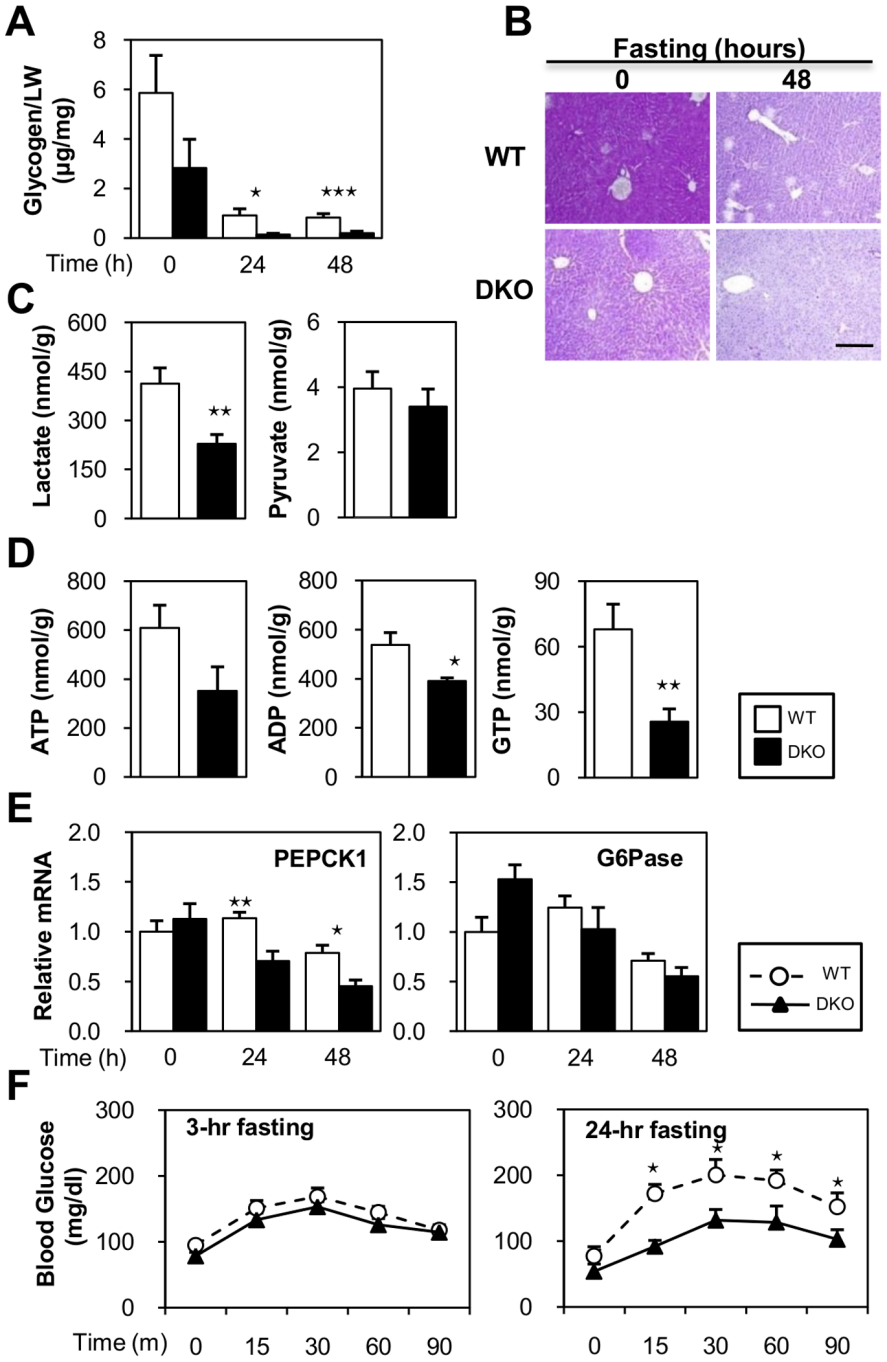
Impaired glucose homeostasis in DKO mice during prolonged fasting. (A to E) Livers were isolated before (0-hr) and after fasting (24- and 48-hr). (A) Glycogen storage in the liver of WT and DKO. n = 8/group (B) Representative PAS staining of the liver of the WT and DKO mice. Scale bar = 100 µm. (C and D) The indicated metabolites from the livers of WT and DKO mice after 48-hr fast were measured using metabolome analysis. n = 7/group. (E) The expression of genes involved in gluconeogenesis was determined using quantitative real-time PCR. (F) Estimated gluconeogenesis using the pyruvate challenge test. After 24 hour of fasting, pyruvate (2 gram/kg) was intraperitoneally injected. Blood was taken from the tail vein to measure the blood glucose levels at the indicated time points. n = 6/group. Data are shown as the mean ± SE. WT vs. DKO. ⋆p<0.05, ⋆⋆p<0.01, ⋆⋆⋆p<0.001.

We next addressed the effects of FABP4/5 deletion on carbohydrate metabolism. Glycogen content in the liver was more remarkably reduced in DKO mice during prolonged fasting (figure 7A), suggesting an accelerated breakdown of glycogen against lower blood glucose levels. Lower glycogen storage during fasting was also confirmed using PAS staining (Figure 7B). An increased level of serum glucagon (figure 1) further supported this hypothesis of accelerated glycogen utilization.

During fasting, glucose is generated from non-carbohydrate precursors, such as lactate and amino acids, mainly in the liver during gluconeogenesis[18]–[21]. Although lactate and amino acids (specifically alanine) maybe supplied from red blood cells and muscle, such substrates present in the liver are also utilized for gluconeogenesis. Metabolome analysis revealed that most precursors in gluconeogenesis (lactate and glucogenic amino acids, such as alanine, asparagine and serine) were significantly reduced after a 48-hr fast (figure 7C and table 1), which strongly suggested that more abundant energy substrates were consumed in the livers of DKO mice via accelerated gluconeogenesis. One exception was glutamate, a central hepatic amino acid, which demonstrated higher levels in the DKO liver. Because glutamate plays a role in the transamination/deamination process and enters into the TCA cycle as α-ketoglutarate [19], it can be increased as an intermediate in these pathways. An increase in glutamate concentration during fasting suggests that facilitation of the glutamate synthesis process, or breakdown of other amino acids for gluconeogenesis, was enhanced at least transiently. An increase in the glucagon/insulin ratio in DKO mice (figure 1) further suggested that DKO mice required more gluconeogenesis. Moreover, metabolome analysis showed that key molecules for gluconeogenesis, such as NAD, NADH and guanosine triphosphate (GTP), were more markedly reduced in the DKO liver (figure 5B and 7D). GTP is usually converted from ATP by a nucleoside disphosphate kinase reaction. ADP, a precursor for ATP, was also reduced in the DKO liver. A reduction in GTP as well as ADP may reflect the diminished ATP synthesis rate during prolonged fasting.

The expression of a key enzyme for gluconeogenesis, phosphoenolpyruvatecarboxykinase (PEPCK1), was comparable prior to fasting, although it was significantly lower in the DKO liver after a 24- and 48-hr fast (figure 7E). These findings suggested that the basal ability of gluconeogenesis was comparable in DKO mice during a short period of fasting, but continued to decline during fasting.

To directly examine the extent of gluconeogenesis, a pyruvate challenge test was performed to measure the blood glucose in response to intra-peritoneal administration of pyruvate (a major gluconeogenic substrate). As expected, DKO mice were compromised in the pyruvate challenge after 24-hr fast (figure 7F). However, the level of gluconeogenesis was comparable after a 3-hr fast (figure 7F), suggesting that materials for gluconeogenesis were available shortly after fasting. Taken together, reduced gluconeogenesis in the DKO liver during prolonged fasting was likely due to a marked reduction in gluconeogenic materials such as lactate, amino acids, GTP, NAD and NADH via accelerated consumption.

### Ketogenesis is Accelerated by the Enhanced Expression of Ketogenesis-associated Genes

**Figure 8:**
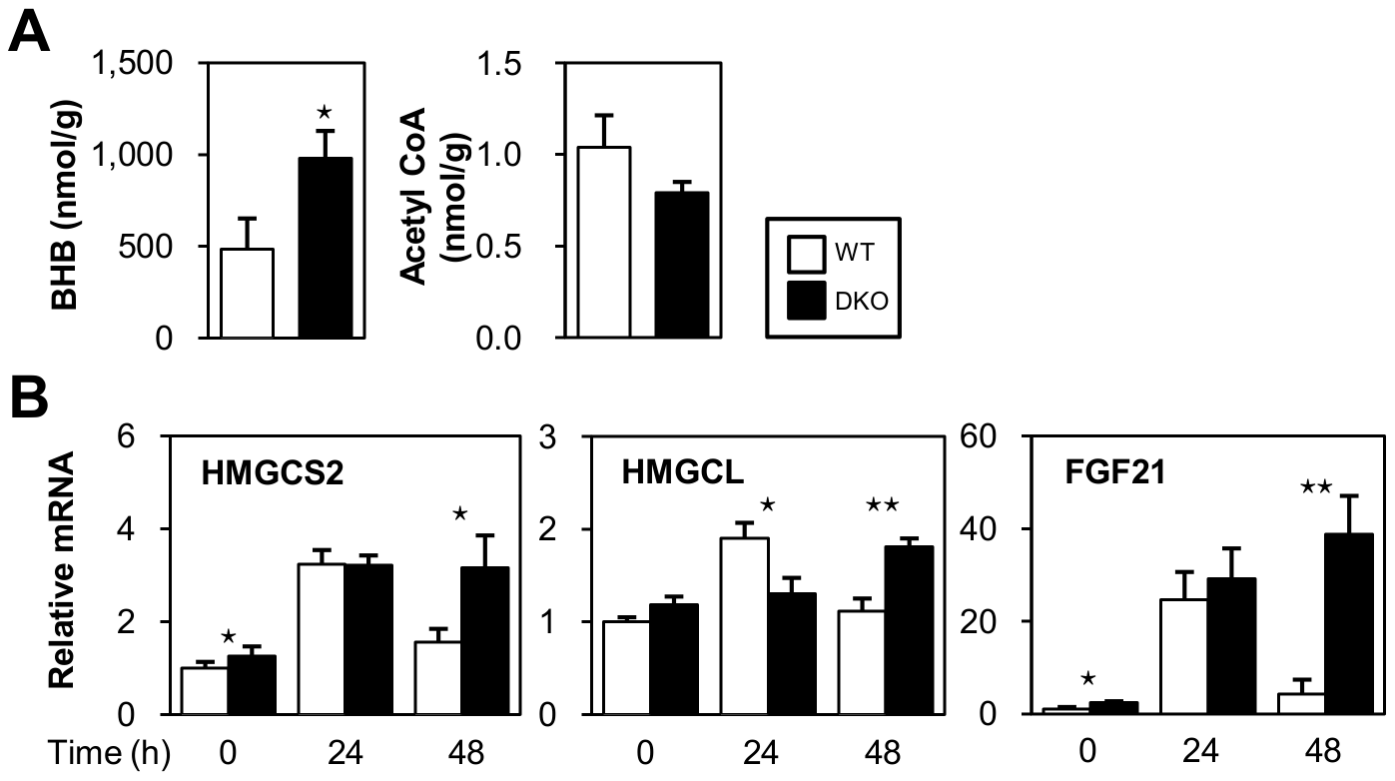
Ketogenesis is enhanced in DKO mice. (A) The indicated metabolites from the livers of WT and DKO mice after 48 hours of fasting were measured using metabolome analysis. n = 7/group. (B) The expression of genes involved in ketogenesis was determined using quantitative real-time PCR. n = 4/group. WT vs.DKO. ⋆p<0.05, ⋆⋆p<0.01.

We found the serum level of BHB was higher in DKO mice during fasting (figure 1). Consistent with this finding, the level of BHB was higher in the DKO liver while the level of acetyl-CoA, a material for ketogenesis, was comparable (figure 8A). RT-PCR further revealed that the expression of genes for ketogenesis, such as 3-hydroxy-3-methylglutaryl-CoA synthase 2 (HMGCS2) and 3-hydroxymethyl-3-methylglutaryl-CoA lyase (HMGCL), were remarkably higher in DKO livers after a 48-hr fast (Figure 8B). In addition, the expression of fibroblast growth factor 21 (FGF21), which is associated with ketogenesis as a direct target gene of PPARα in response to fasting [22], [23], was markedly higher in DKO livers after a 48-hr fast (Figure 8B). These findings suggested that the enhanced expression of these ketogenic genes during fasting may at least partially accelerate ketogenesis in DKO mice.

## Discussion

**Figure 9:**
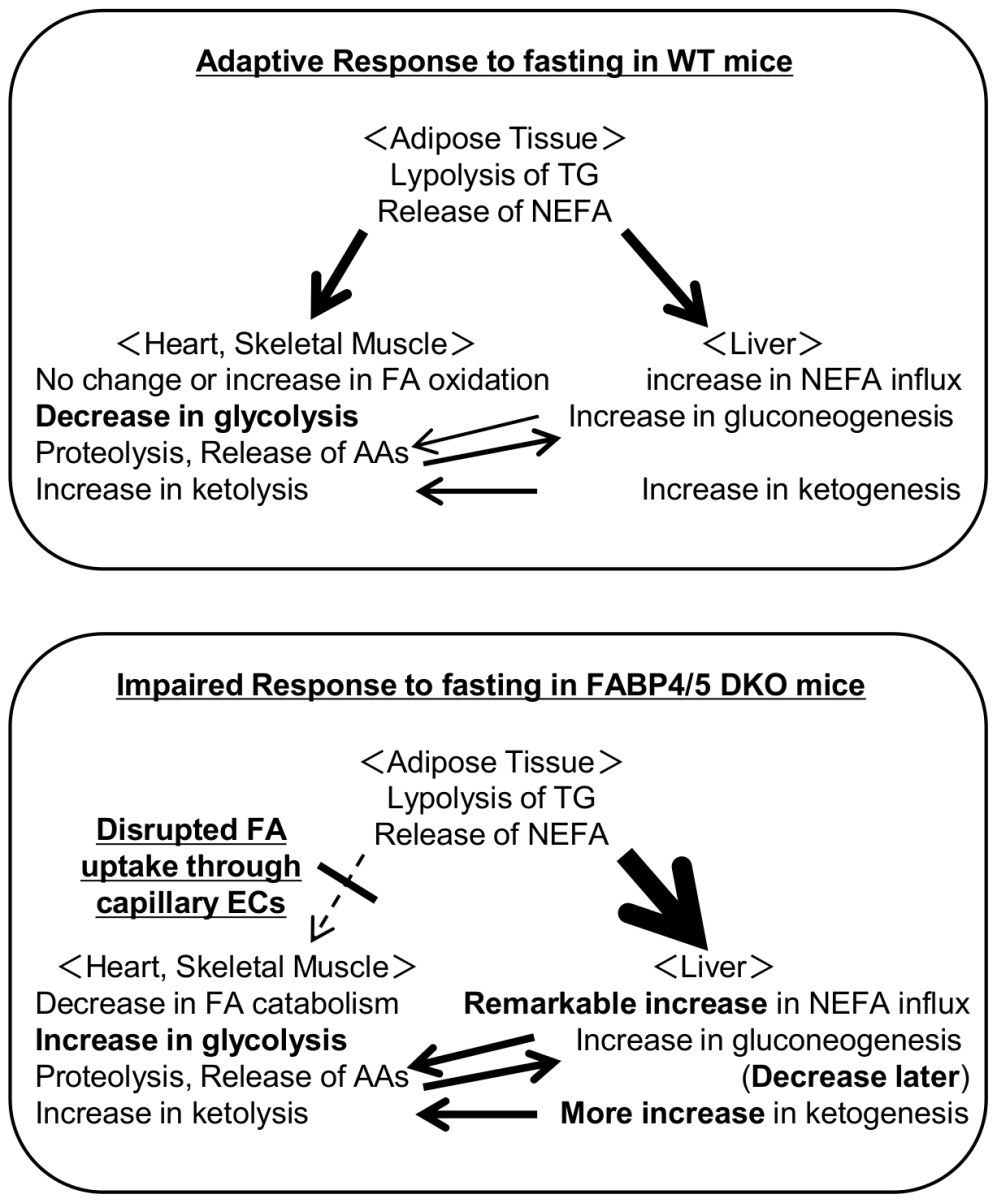
Working model of metabolic changes in DKO mice during fasting. (A) In WT mice, TG in adipose tissue is hydrolyzed during prolonged fasting, which releases NEFA into circulation. NEFA is taken up by various organs, including the heart, skeletal muscle and the liver as central energy substrates, which spares glucose consumption for glucose-dependent tissues, such as the brain and red blood cells. (B) However, in DKO mice, circulating NEFA cannot be efficiently taken up by the heart and skeletal muscle due to impaired FA transport via capillary ECs, which results in an increase in NEFA influx into the liver and FA accumulation in the liver. To compensate for the reduced uptake of NEFA, glucose uptake by the heart and red skeletal muscle is markedly enhanced independently of insulin even during fasting [14], which causes hypoglycemia. Although gluconeogenesis is conserved to supply glucose to peripheral tissues shortly after fasting, substrates for gluconeogenesis are reduced, resulting in insufficient gluconeogenesis during prolonged fasting, which further enhances hypoglycemia. FAO was enhanced during the fed state and a higher level of FAO was maintained even after prolonged fasting. Combined metabolic changes, including increased NEFA influx into the liver, enhanced FAO and lower blood glucose, accelerate ketogenesis. Please refer to the text and discussion for further details. Thick arrows indicate more flow; thin arrows indicate less flow; dotted arrow indicates impaired flow.

Our study established that FABP4/5 play a critical role in the systemic response to prolonged fasting. The major findings of this study included the following: first, the blood glucose concentration was significantly lower while serum levels of NEFA and ketone bodies were significantly higher during prolonged fasting in DKO mice (hyperketotic hypoglycemia). Second, prolonged fasting markedly induced hepatic steatosis in DKO mice. Third, FAO was enhanced during the fed state, and higher levels of FAO were maintained even after prolonged fasting. Fourth, gluconeogenesis was markedly reduced after prolonged fasting. Consistent with our recent observations, these results indicated that a systemic change in metabolism was likely caused by a reduction in the FA uptake in the heart and skeletal muscle as well as compensatory glucose consumption in these tissues, thus linking endothelial FABP4/5 to the regulation of systemic energy homeostasis during the fasting condition (figure 9).

### Distinct Mechanisms of Hepatic Fat Accumulation in DKO Mice

The potential mechanisms contributing to the excessive accumulation of fat in the liver include increased fat delivery, increased fat synthesis, reduced fat oxidation, and/or reduced fat export in the form of VLDL. Among these mechanisms, hepatic steatosis in DKO mice is likely to be largely attributed to increased fat delivery into the liver. Hepatocytes can take up to 30% of circulating NEFA, which may exceed its capacity for FA [19]. Thus, increased levels of circulating NEFA, as observed in DKO mice during fasting, may be one of the major causes of prominent fatty liver. In addition, the uptake of ^125^I-BMIPP by the liver was increased in DKO mice during fasting, which further supports this hypothesis. Our study using tyloxapol, a LPL inhibitor, showed that VLDL secretion in the liver was diminished in DKO livers after 24-hr fast, which was consistent with the reduced expression of genes associated with VLDL formation, such as Apo-B100. In addition, we showed an increased level of serum glucagon, which may further facilitate steatosis due to glucagon suppression of VLDL secretion [17]. Our data excluded the possible contribution of reduced FAO because the FAO rate by liver extracts was significantly increased in DKO mice compared to WT mice. Taken together, these data indicate that an increase in circulating NEFA and a reduced fat export in the form of VLDL contribute to hepatic steatosis in DKO mice during prolonged fasting.

### Mechanisms Underlying Pronounced Hypoglycemia during Fasting in DKO Mice

One of the salient features of the metabolism in DKO mice was pronounced hypoglycemia compared to WT mice during prolonged fasting. Extreme hypoglycemia maybe attributed to two major causes; remarkable glucose consumption by peripheral tissues and reduced gluconeogenesis. Glucose uptake is accelerated in FA-burning tissues, such as the heart and red skeletal muscle, to compensate for restricted FA utilization due to impaired FA uptake via capillary endothelial cells [14]. The dramatic increase in glucose utilization during fasting was insulin-independent and was at least partially attributed to the post-transcriptional and allosteric regulation of key proteins that regulate glucose uptake and glycolysis [14]. Although gluconeogenesis was conserved at a 3-hr fast, it was significantly reduced at a 24-hr fast. Metabolome analysis revealed that gluconeogenic materials such as lactate, most amino acids and co-factors (NAD, NADH and GTP) were markedly reduced in DKO livers after prolonged fasting. An increase in glutamate concentration and an increase in the glucagon/insulin ratio in DKO mice during fasting suggested that gluconeogenesis was accelerated at least transiently. Taken together, gluconeogenesis was most likely accelerated to maintain the glucose levels shortly after fasting, which resulted in lower levels of gluconeogenic substrates during prolonged fasting, thereby leading to a reduced ability of subsequent gluconeogenesis at later time periods. However, it is still uncertain whether the acceleration of gluconeogenesis and/or other mechanisms was directly related to a decrease in gluconeogenic substrates. Our data suggested that elevated glucose consumption by peripheral tissues as well as the subsequent reduction of gluconeogenesis caused marked hypoglycemia during fasting.

### Hyperketonemia is a Unique Feature in DKO Mice

The mechanisms of increased levels of circulating ketone bodies in DKO mice deserve further discussion. Since FAO is elevated in the liver of DKO mice independent of fasting (figure 5D), an abundant supply of NEFA into hepatocytes (figure 1 and 2E) and a lower level of blood glucose during fasting (figure 1) likely promoted ketogenesis (figure 8) in combination with an enhanced expression of genes involved in ketogenesis (figure 7B). Hyperketonemia is a unique feature in DKO mice with defective peripheral FA utilization when compared with other mice lacking FA utilization. Mice deficient for FAO-associated genes, such as MCAD, LCAD and PPARα, showed metabolic changes that were very similar to DKO mice in response to fasting, such as marked steatosis, hypoglycemia and an elevated level of serum NEFA[1]–[4]. However, they did not have hyperketonemia, but did exhibit hypoketonemia or normoketonemia. Although FA utilization was disrupted in the heart and red skeletal muscle at the capillary level in DKO mice, FAO was conserved or even increased in the liver. However, in mice lacking FAO-associated genes, FAO was disrupted at different steps of the pathway and to varying degrees in most tissues, including the liver. Moreover, NEFA was efficiently converted into ketone bodies without functional disturbance of FAO in DKO livers, whereas most of the NEFA accumulated as TG without efficient oxidation in the liver in mice deficient for FAO-associated genes. Thus, our data demonstrated that DKO mice exhibited distinct features of ketone body metabolism and hyperketonemia, which was in sharp contrast to the hypoketonemia/normoketonemia reported in mice lacking FAO-associated genes, where common characteristics such as marked steatosis, hypoglycemia and an elevated level of serum NEFA were observed.

In summary, we showed that a loss of FABP4/5 resulted in hyperketotic hypoglycemia and marked hepatic steatosis during prolonged fasting. Together with our previous study demonstrating that DKO mice exhibit defective FA uptake in the heart and red skeletal muscle across capillary endothelial cells and a compensatory increase in glucose uptake in these tissues, the results in the present study established FABP4/5 as important components of the systemic adaptive response to fasting, and may have important implications for understanding the metabolic consequences of defective peripheral FA uptake, as well as for developing a novel strategy to control energy homeostasis via the selective modulation of FA uptake.

## Materials and Methods

### Mice and Sample Collection

Mice with a homozygous null mutation in *Fabp4* (*Fabp4*−/−) or *Fabp5* (*Fabp5*−/−) were generated as previously described [6], [7]. Mice deficient for both *Fabp4* and *Fabp5* (DKO mice) were generated from an intercross between *Fabp4*(−/−) and *Fabp5*(−/−) mice as previously described [9]. The background strain of the wild-type and knockout mice was C57BL6J. The Institutional Animal Care and Use Committee (Gunma University Graduate School of Medicine) approved all study protocols. The mice were housed in a temperature-controlled room in a 12-hour light/12-hour dark cycle and had unrestricted access to water and standard chow (CE-2, Clea Japan, Inc.). Twelve to eighteen week-old mice from both genotypes were used. For the fasting experiments, the mice were individually housed and food was withdrawn for 24 or 48 hr; water was provided *ad libitum*. A portion of the liver was snapped frozen in liquid nitrogen and kept at −80°C until further use. The rest of the liver was processed for histological examination. Locomotor activity was measured using the ACTIMO-100 (SHINFACTORY, Fukuoka, Japan).

### Measurement of Blood Parameters

Blood was collected from the vena cava inferior and centrifuged at 1,500×*g* for 15 minutes at 4°C to separate the serum. Blood glucose was measured using the glutest sensor (Sanwa Kagaku, Aichi, Japan). Serum levels of insulin (Ultrasensitive Mouse Insulin ELISA, Mercodia, Uppsala, Sweden), ketone bodies (EnzyChrom Ketone Body Assay Kit, BioAssaySystems, California), triglycerides (Triglyceride E-test, Wako Chemical, Osaka), non-esterified fatty acids (NEFA C-test, Wako Chemical, Osaka) and total cholesterol (Cholesterol E-test, Wako Chemical, Osaka) were measured according to the manufacturer’s protocols. Blood for glucagon measurement was separately collected from the retro-orbital plexus and transferred into a tube containing aprotinin (500 KIU/ml). Glucagon was measured according to the manufacturer’s protocol (Glucagon RIA kit, Millipore, USA).

### Triglyceride Measurements in the Liver

Livers were homogenized in RIPA buffer (50 mM Tris-HCl, pH 7.4, 1% NP40, 0.25% Na-deoxycholate, 150 mM NaCl and 1mM EDTA) and centrifuged at 18,000×*g* for 10 minutes at 4°C. Lipids in the supernatant were extracted with methanol/chloroform (1∶2), evaporated with NO_2_ and dissolved in isopropanol. Triglyceride contents were determined using the Triglyceride E-test Wako (Wako Chemical, Osaka) and were expressed as milligrams of lipid per gram of protein.

### Glycogen Measurements in the Liver

Liver samples were immediately snap-frozen in liquid nitrogen and pulverized using a mortar and pestle in liquid nitrogen. Ten milligrams of liver powder was resuspended in distilled water and then boiled for 5 minutes. After centrifugation, the supernatants were collected to measure the glycogen concentration (BioVision).

### Histological Analysis

For oil red O stain, the liver was fixed with 4% paraformaldehyde, embedded in OCT compound (Tissue-Tek, Netherlands) and frozen in liquid nitrogen. Cryosections (10 µm) were immersed for 10 minutes in 0.3% oil red O in isopropanol. For PAS staining, the liver was fixed with 4% paraformaldehyde and embedded in paraffin.

### RNA Isolation and Reverse Transcription (RT)–PCR

Total RNA was isolated from various organs using TRIzol reagent (Invitrogen). Semi-quantitative RT-PCR was performed using the RT-PCR kit (TAKARA, Japan) according to the manufacturer’s protocol. Quantitative real time-PCR was performed with the SYBR Green PCR Master Mix (Applied Biosystems) according to the manufacturer’s instructions. The expression level of the target gene was normalized against GAPDH mRNA levels. The gene-specific primers for cDNA used in this study are listed in table 2.

**Table 2:** Primer for RT-PCR.

	Forward	Reverse
mACC	GAATCCTCATTGGCTTACGATGAG	AATGGCCCGGCATGT
mACSL1	GAACAGAGTGAAGCCCAAGC	CAGGCTGTTTCTGACGA
mApoB100	CGTGCAAGAACTGGCTGATA	CAGCGCTCCAAGTGACATTA
mCPT1a	CCATGAAGCCCTCAAACAGATC	ATCACACCCACCACCACGATA
mCPT2	TCCTCGATCAAGATGGGAAC	GATCCTTCATCGGGAAGTCA
mELOVL6	CCCGAACTAGGTGACACGAT	TACTCAGCCTTCGTGGCTTT
mFABP1	CATCCAGAAAGGGAAGGACA	TTTTCCCCAGTCATGGTCTC
mFAS	GCTGCGGAAACTTCAGGAAAT	AGAGACGTGTCACTCCTGGACTT
mFAT/CD36	GAGCAACTGGTGGATGGTTT	TGGGTTTTGCACATCAAAGA
mFATP2	GGTATGGGACAGGCCTTGCT	GGGCATTGTGGTATAGATGACATC
mFGF21	CTGGGGGTCTACCAAGCATA	GTCCTCCAGCAGCAGTTCTC
mG6Pase	CCAGTCGACTCGCTATCTCC	CAAGGTAGATCCGGGACAGA
mGAPDH	AGCCCCCAGTCTGTATCCTT	TCCACCACCCTGTTGCTGTA
mHMGCL	ACTACCCAGTCCTGACTCCAA	TAGAGCAGTTCGCGTTCTTCC
mHMGCS2	ATACCACCAACGCCTGTTATGG	CAATGTCACCACAGACCACCAG
mPEPCK1	CGATGACATCGCCTGGATGA	TCTTGCCCTTGTGTTCTGCA
mPPARα	AGAGCCCCATCTGTCCTCTC	ACTGGTAGTCTGCAAAACCAAA
mSCD1	GCTGGGCAGGAACTAGTGAG	GGTAGGGAGGATCTGGAAGC

RT PCR indicates Real Time Polymerase Chain Reaction; ACC, acetyl CoA aarboxylase; ACSL1, acyl-CoA synthetase long-chain family member 1; ApoB100, apolipoprotein B100; CPT, carnitinepalmitoyltransferase; FABP1, fatty acid binding protein 1; FAS, fatty acid synthase; FAT, fatty acid translocase; FATP2, fatty acid transport protein 2; FGF21, fibroblast growth factor 21; G6Pase, glucose-6-phosphatase; GAPDH, glyceraldehyde-3-phosphate dehydrogenase, HMGCL, 3-hydroxymethyl-3-methylglutaryl-CoA lyase; HMGCS2, 3-hydroxy-3-methylglutaryl-CoA synthase 2; PEPCK1, phosphoenolpyruvatecarboxykinase 1, PPARα, peroxisome proliferator-activated receptor α, SCD1, stearoyl-coA desaturase 1.

### Isolation of Hepatocytes from Mice

Primary cultures of hepatocytes from adult mice were prepared as previously described by Li et al. with slight modifications [24]. Under anesthetized conditions, the liver was perfused with perfusion medium I (10 mM HEPES, 0.05% KCl, 5 mM glucose and 0.2 mM EDTA, in phosphate-buffered saline [PBS] at pH 7.4) from the vena cava using a peristaltic pump (5 ml/min) for 8 minutes to the portal vein, which was cut after needle insertion. Next, perfusion medium II (30 mM HEPES, 0.05% KCl, 5 mM glucose and 1 mM CaCl_2_ in PBS at pH 7.4) containing 0.8 mg/ml of collagenase L (Nitta Gelatin) was used to digest the tissue for 8 minutes. After digestion and removal of the gall bladder and unrelated tissues, the liver was minced into small pieces and filtered into a new tube via a gauze mesh filter. Hepatocytes were centrifuged at 50×*g* for 2 minutes and washed with perfusion medium II three times, and then re-suspended with hepatocyte medium (DMEM high glucose containing 2 mM glutamine, 1% non-essential amino acids, 10% fetal bovine serum, 1% penicillin/streptomycin, 50 µg/ml of gentamycin. After an overnight incubation, the ^14^C-Palmitic acid uptake assay or β-oxidation assay was performed as described below.

### ^14^C-Palmitic Acid Uptake by Cultured Hepatocytes

Hepatocytes were plated in 6-well plates at density of 1 ×10^5^ cells and pre-incubated in serum-free DMEM (Invitrogen) for thirty minutes. To determine the dose-related uptake, hepatocytes were incubated with 0.18 kBq (low dose) and 3 kBq ^14^C-palmitic acid (high dose) with FA-free bovine serum albumin (BSA) as a carrier. After 10 minutes, the cells were washed with ice-cold stop buffer (0.1% BSA and 0.2 mM phloretin in PBS) three times and lysed in lysis buffer (0.1 N NaOH, 0.2% SDS). Supernatants were collected into tubes containing 4 ml of scintillation cocktail (Aquasol2; Perkin Elmer). The radioactivity of the cell lysate was determined using the liquid scintillation counter (LCS-3000; Aloka). The experiments were performed independently three times.

### Palmitic Acid Oxidation Studies in Cultured Hepatocytes

Fatty acid oxidation in primary culture of hepatocytes was assessed by measuring the production of ^14^CO_2_as previously described [25]. Briefly, isolated hepatocytes were plated in a T25 flask at a density of 1 ×10^6^ cells and pre-incubated in glucose-free DMEM (Invitrogen) for thirty minutes. The cells were treated with ^14^C-palmitic acid (Perkin Elmer, CT, USA) with FA-free BSA as a carrier and a piece of wet Whatman filter paper with 2N sodium hydroxide was suspended within each flask. The flasks were sealed, and 7 hours later, the cells were lysed in 6N hydrochloric acid and then further incubated overnight. The ^14^CO_2_ produced was trapped on the Whatman paper and was quantified using scintillation counting.

### NEFA β Oxidation in Liver Homogenates

The beta oxidation ability of liver homogenates was examined as previously described [17]. One hundred milligrams of liver was manually homogenized using a Potter-Elvejhem homogenizer in 2 ml of ice-cold isolation buffer (220 mM mannitol, 70 mM sucrose, 2 mM HEPES, and 0.1 mM EDTA at pH 7.4). Aliquots of the homogenate (300 µl) were added to 1.7 ml of reaction medium (50 mM sucrose, 150 mM Tris-HCl, 20 mM KH_2_PO_4_, 10 mM MgCl_2,_ 2 mM EDTA, 1 mM L-carnitine, 0.2 mM CoA, 2 mM NAD, 0.1 mM malate, 10 mM ATP, 1 mM palmitate complexed to fatty acid-free albumin at a 5∶1 molar ratio in Williams E medium, 12 kBq of ^14^C-palmitic acid [Perkin Elmer, CT, USA], pH 7.4). The reactions were performed in a sealed flask and were placed in a 37°C incubator for 30 minutes. The incubations were terminated by the addition of 1 ml of 6N hydrochloric acid to the reaction medium to precipitate proteins and non-metabolized palmitate. The sample was further incubated at room temperature for 2 hours to collect ^14^CO_2_ into a suspended well containing 250 µl of ethanolamine. Blanks were prepared by acidification of the reaction medium immediately after the addition of the homogenate. Radioactivity in ^14^CO_2_ was quantified using liquid scintillation counting.

### Biodistribution of ^125^I-BMIPP (15-(p-iodophenyl)-3-(R,S)-methyl Pentadecanoic Acid) and ^18^F-FDG (2-fluorodeoxyglucose)

The biodistribution of ^125^I-BMIPP and ^18^F-FDG was determined as previously described[26]–[28]. Mice received intravenous injections of ^125^I-BMIPP (5 kBq) and ^18^F-FDG (100 kBq) via the lateral tail vein in a volume of 100 µl.^ 125^I-BMIPP was a gift from Nihon Medi-Physics Co. Ltd. and ^18^F-FDG was obtained from batches prepared for clinical PET imaging at Gunma University. The animals were sacrificed 2 hours after the injection. The isolated tissues were weighed and quantified using a well-type gamma counter (ARC-7001, ALOKA). Each experiment was performed at least twice.

### Pyruvate Challenge Test

After 3- or 24-hr fast, pyruvate (2 gram/kg body weight, Sigma) was injected intraperitoneally. Blood was taken from the tail vein to measure the level of glucose using the glutest sensor (Sanwa Kagaku, Aichi, Japan) at the indicated time points [29].

### Hepatic TG-VLDL Production

After 24 hours of fasting, the mice were intravenously injected in the tail vein with Triton WR 1339 (500 mg/kg body weight in 0.9% NaCl), which inhibits the enzymatic activity of lipoprotein lipase [30]. Blood was sampled before (0 hour) and after (1, 2 and 6 hours) the injection. TG was measured enzymatically as previously described. The TG-VLDL production rate was calculated from the difference in plasma TG levels over a given interval following detergent injection and was expressed as mmol/kg/h as previously reported [30].

### Metabolome Analysis Using CE-MS

Metabolome analyses were performed as described elsewhere [31]. The abdomen of the mouse was opened by a midline incision under isoflurane anesthesia, which did not stop breathing and the heart beat. A portion of the liver was rapidly removed from the mice at 3 months of age after a 48-hr fast. The time between isolating the liver and snap-freezing it in liquid nitrogen was within several seconds and the tissue was stored at −80°C.

#### Metabolite extraction

Frozen liver tissue was immediately immersed into methanol (1 ml) containing internal standards (300 µM each of methionine sulfone for cations and MES for anions) and homogenized for 1 minute to inactivate enzymes. Next, deionized water (500 µl) was added, and 300 µl of the solution was transferred into another tube. Subsequently, 200 µl of chloroform was added, and the mixture was thoroughly mixed. The solution was then centrifuged at 12,000×*g* for 15 minutes at 4°C, and 300 µl of the upper aqueous layer was centrifugally filtered through a Millipore 5-kDa cutoff filter to remove proteins. The filtrate was lyophilized and dissolved in 50 µl of Milli-Q water containing the reference compounds (200 µM each of 3-aminopyrrolidine and trimesate) prior to CE-MS analysis.

#### Metabolic standards

All of the chemical standards were obtained from common commercial sources and dissolved in Milli-Q (Millipore) water, 0.1 N HCl or 0.1 N NaOH to obtain 10 mM or 100 mM stock solutions. Working standard mixtures were prepared by diluting the stock solutions with Milli-Q water just prior to the injection into the CE-MS. The chemicals used were of analytical or reagent grade.

#### Instrument

All CE-MS experiments were performed using the Agilent CE Capillary Electrophoresis System equipped with an air pressure pump, the Agilent 1100 series MSD mass spectrometer, and the Agilent 1100 series isocratic high performance liquid chromatography pump, a G1603A Agilent CE-MS adapter kit, and a G1607A Agilent CE-MS sprayer kit (Agilent Technologies). System control, data acquisition, and MSD data evaluation were performed using the G2201AA Agilent Chem Station software for CE-MSD.

#### CE-MS conditions for cationic metabolites

Separations were performed in a fused silica capillary (50 µm inner diameter x 100 cm total length) filled with 1 M formic acid as the electrolyte. Approximately 3 nl of the sample solution was injected at 50 mbar for 3 seconds, and the voltage was applied at 30 kV. The ESI-MS was performed in the positive ion mode, and the capillary voltage was set at 4000 V. For MS, using the selective ion monitoring mode, deprotonated [M+H]^+^ ions were monitored for cationic metabolites of interest.

#### CE-MS conditions for anionic metabolites

A cationic polymer-coated SMILE (+) capillary was obtained from Nacalai Tesque (Kyoto, Japan) and used as the separation capillary (50 µm inner diameter×100 cm total length). The electrolyte for the CE separation was 50 mM ammonium acetate solution (pH 8.5). The samples were injected with a pressure injection of 50 mbar for 30 seconds (30 nl). The applied voltage was set at -30 kV. ESI-MS was performed in the negative ion mode, and the capillary voltage was set at 3500 V. For MS, using the selective ion monitoring mode, deprotonated [M–H]^−^ ions were monitored for anionic metabolites of interest.

### Statistical Analysis

Statistical analysis was performed using unpaired Student’s t-test for 2 samples. One way ANOVA was performed for 3 samples and Bonferroni’s post hoc multiple comparison tests were performed to evaluate differences between the control and experimental groups. A p-value of <0.05 was considered statistically significant.
